# Glycogen Synthase Kinase 3β Promotes Osteogenic Differentiation of Murine Adipose-Derived Stromal Cells

**DOI:** 10.1371/journal.pone.0054551

**Published:** 2013-01-16

**Authors:** Jeong-Eun Huh, Ryeojin Ko, Hyun Ju Jung, Soo Young Lee

**Affiliations:** 1 Division of Life and Pharmaceutical Sciences, Ewha Womans University, Seoul, Korea; 2 Department of Bioinspired Science, Department of Life Science, Ewha Womans University, Seoul, Korea; University of Minnesota Medical School, United States of America

## Abstract

Although the role of glycogen synthase kinase 3β (GSK3β) in osteogenic differentiation of bone marrow-derived mesenchymal stromal cells (BMSCs) is well-characterized as a negative regulator of β-catenin, its effect on osteogenesis of adipose-derived stromal cells (ADSCs) is poorly understood. Here, we show that GSK3β positively regulates osteogenic differentiation of murine ADSCs. Gain-of-function studies showed that GSK3β promotes in vitro osteogenesis of ADSCs. Regulation of GSK3β activity in ADSCs, either by small interfering RNA (siRNA)-mediated GSK3β silencing or by pharmacological inhibitors, blunted osteogenesis and the expression of osteogenic markers. Importantly, we demonstrated that transgenic mice, engineered to overexpress the constitutively active GSK3β (GSK3β-S9A) mutant, exhibited a marked increase in osteogenesis, whereas expression of the catalytically inactive GSK3β (GSK3β-K85A) in mice inhibits osteogenic differentiation. Molecular analyses showed that the enhanced osteoblast differentiation induced by GSK3β was mediated by downregulation of β-catenin. Remarkably, β-catenin silencing enhances osteogenesis and osteoblast marker gene expression such as alkaline phosphatase (ALP) and osterix. Taken together, these findings demonstrate a novel role for GSK3β in the regulation of osteogenic differentiation in ADSCs.

## Introduction

Adipose-derived stromal cells (ADSCs) represent a readily available abundant supply of mesenchymal stem cells [1]. ADSCs are simply expanded to large numbers in vitro, when compared with bone marrow-derived mesenchymal stromal cells (BMSCs) [2] and there is less cell heterogeneity in ADSCs than there is in BMSCs due to the mixture of hematopoietic and mesenchymal stem cells [3], [4]. Similar to BMSCs, ADSCs can differentiate into osteoblasts, adipocytes, and chondrocytes by different inductive culture systems [5]–[10]. Although BMSCs are considered as a valuable source for bone tissue regeneration in human diseases [11], [12], the capacity of autologous BMSCs to differentiate along functional bone-forming osteoblasts remains relatively limited for bone regeneration in vivo [13]. An important issue for efficient bone regeneration is therefore to make ADSCs a promising source of skeletal progenitor cells, to promote their osteogenic potential for in vivo bone regeneration [14]. In this regard, the regulatory mechanism of osteogenesis, and ways to improve osteodifferentiation of ADSCs need to be determined in detail.

Osteogenesis is defined by a series of events, which starts with a commitment to an osteogenic lineage by mesenchymal cells. Subsequently, these cells proliferate and demonstrate an upregulation of osteoblast-specific genes and mineralization [1]. Multiple signaling pathways have been demonstrated to participate in the differentiation of an osteoblast progenitor to a committed osteoblast, including transforming growth factor β/BMP, Wnt/β-catenin, Notch, fibroblast growth factor, and Hedgehog [15]–[25]. Wnts, in particular, have been demonstrated to play a significant role in either embryonic development or osteoblast differentiation [17]–[19]. Glycogen synthase kinase 3β (GSK3β) is a key component of the canonical Wnt signaling pathway [26]–[28]. GSK3 phosphorylates β-catenin, and phosphorylated β-catenin is subjected to ubiquitin proteasome degradation. However, upon Wnt binding to its receptors, frizzled and low-density lipoprotein receptor-related protein β-catenin phosphorylation by GSK3β is inhibited, and β-catenin is stabilized. Stabilized β-catenin translocates into the nucleus and induces target gene expression. Although GSK3β in osteogenesis of BMSCs is well-characterized as a negative regulator of β-catenin, its effect on osteogenesis of ADSCs is not established.

In this study, we found that GSK3β in ADSCs has a positive effect on osteogenic differentiation. We uncovered that GSK3β-mediated β-catenin protein levels determine the osteogenic capacity of ADSCs.

## Materials and Methods

### Ethics statement

All animal experiments were done with the approval of the ethical committees at the Ewha Womans University.

### Cell culture and reagents

ADSCs were isolated from 6 to 8-week-old C57BL/6 mice (The Jackson Laboratory) as described previously [29]. In brief, adipose tissue, dissected from mouse inguinal and lateral abdominal fat and cut into fine pieces, was digested with 0.075% collagenase IA (Sigma) for 1 hour at 37°C with vigorous shaking. The released cells were centrifuged at 300×*g* for 10 minutes and the cell pellet, suspended with phosphate-buffered saline (PBS), was filtered through a 100 μm cell strainer (BD Biosciences, San Jose, CA) to remove tissue debris. Collagenase was removed by dilution with PBS and centrifuged twice at 300×g for 10 minutes. The cell pellet was suspended in 0.83% NH_4_Cl and incubated to remove contaminating red blood cells. The residual cells were washed and centrifuged twice with PBS under same conditions, and cultured in Dulbecco's modified Eagle's medium (DMEM; HyClone, Logan, UT) supplemented with 10% fetal bovine serum (FBS; HyClone), 100 units/ml penicillin, and 100 μg/ml streptomycin (HyClone) in tissue culture dishes at 37°C in a 5% CO_2_ humidified incubator. After 4 hours, non-adherent cells were removed by two to three washes with PBS, and adherent cells further cultured in complete medium until the cells reached 80 to 90% confluence. ADSCs from passage 3 to passage 5 were used in all experiments described. HEK293T cells were maintained in Dulbecco's modified Eagle's medium (DMEM; HyClone) with 10% FBS and antibiotics. All other chemicals including SB216763 and LiCl were purchased from Sigma (St. Louis, MO).

### Osteogenic differentiation and alkaline phosphatase assay

The potential of the isolated cells to differentiate into osteogenic lineages was examined. For osteogenesis, cells were allowed to grow to 70% to 90% confluence and were then cultured in osteogenic medium containing 10 mM β-glycerol phosphate (Sigma), 50 μg/ml ascorbate-2-phosphate (Sigma), 10^−7^ M dexamethasone (Sigma) and 25 ng/ml human recombinant bone morphogenetic protein 2 (BMP-2, R&D Systems). The culture medium was changed three times per week for 1 to 2 weeks, respectively. Alkaline phosphatase (ALP) activity was detected by BCIP/NBT color development substrate (Promega, Wisconsin, USA) according to the manufacturer's instructions.

### Alizarin red S (ARS) and von Kossa (VK) staining

The degree of extracellular matrix calcification was estimated using ARS staining and VK staining. Briefly, cells were fixed with 4% paraformaldehyde for 10 minutes at room temperature and stained with 2% ARS, pH 4 (Alphachem, Middlesex, UK) for 5 minutes at room temperature. For VK staining, cells were fixed with methanol for 10 minutes at room temperature and overlaid with 1% silver nitrate solution (Sigma) under UV light for 1 hour.

### siRNA transfection

Double-stranded, siRNAs (21-mer) targeting mouse GSK3β and β-catenin were synthesized from Genolution Pharmaceuticals Inc. (Seoul, Korea). The corresponding target mRNA sequences for the siRNAs were as follows: si-GSK3β, ACACGAAAGTGATTGGAAA; si-β-catenin, GTTGCTTTGCTCAACAAAA; scrambled nontargeting siRNA, ACGTGACACGTTCGGAGAA, as a negative control. ADSCs were transfected with the gene-specific siRNA at a concentration of 10 nM using Lipofectamine^TM^ RNAiMAX (Invitrogen) according to the manufacturer's protocol.

### Retroviral infection

HA-tagged cDNAs of wild-type GSK3β (GSK3β-WT) and two mutants, catalytically inactive GSK3β (GSK3β-K85A) and constitutively active GSK3β (GSK3β-S9A) cloned into the retroviral vector, pMX-puro were previously described [30]. The plasmids were transfected into Platinum-E (Plat-E) cells using polyethylenimine (PEI; Sigma) reagent, and the supernatant was collected 24 to 36 hours after transfection. The pMX-puro vector and Plat-E cells were kindly provided by T. Kitamura (University of Tokyo, Japan). The packaged retroviral particles in the supernatant were filtered through 0.45 μm filters (BD Biosciences), supplemented with polybrene (10 μg/ml; Sigma) then used to infect ADSCs that had been seeded 24 hours before infection. After 24 hours, 1 μg/ml puromycin was added to the medium to select for infected cells for 2 days. Puromycin-resistant cells were used in all experiments described.

### Western blot analysis

Cells were lysed in cell lysis buffer containing 50 mM Tris-HCl (pH 7.4), 150 mM NaCl, 0.5% sodium deoxycholate, 1% Nonidet P-40, 1 mM EDTA, 10% glycerol, 1 mM phenylmethylsulfonyl fluoride (PMSF), 1 μg/ml of leupeptin, aprotinin, and pepstatin A, 1 mM sodium orthovanadate and 1 mM sodium fluoride (NaF) for 20 minutes on ice. The lysates were centrifuged at 14,000×g for 20 minutes at 4°C. The supernatants were boiled in SDS sample buffer containing 0.5 M β-mercaptoethanol. Cytosol and nuclear protein fractionation was performed using the NE-PER Nuclear and Cytoplasmic Extraction kit (Pierce) according to the manufacturer's instructions. Protein concentration was determined by the Bradford assay (Bio-Rad). Equal amounts of proteins were separated by 10% SDS-PAGE and electrotransferred to a PVDF membrane (Millipore, Billerica, MA, USA). The membrane was blocked in PBS containing 5% nonfat dry milk for 1 hour and then immunoblotted overnight at 4°C on a shaker with antibodies against GSK3α/β (Invitrogen), phospho-Ser^9^-GSK3β, hemagglutinin (HA) (Cell Signaling Technology, Danvers, MA), β-catenin (BD Biosciences), tubulin (Santa Cruz Biotechnology), TATA-binding protein (TBP; Abcam, Cambridge, MA), and glyceraldehyde 3 phosphate dehydrogenase (GAPDH; Abfrontier, Seoul, Korea) in Tris-buffered saline containing 0.05% tween 20 (TBST) with 1% bovine serum albumin (BSA). Following wash with TBST, the membrane was incubated with either horseradish peroxidase-conjugated anti-rabbit antibody or anti-mouse antibody (Thermo Scientific) in TBST. Proteins were detected using an ECL detection kit (Amersham Biosciences, NJ, USA).

### Reverse transcription PCR (RT-PCR) and real time PCR

Total RNA was isolated from ADSCs using TRIzol reagent (Invitrogen) according to the manufacturer's instructions. After denaturation of total RNA at 70°C for 10 minutes, first-strand cDNA were synthesized with oligo (dT) primers and MMLV-reverse transcriptase (SolGent, Seoul, Korea). PCR amplifications were performed using the specific primers. Primer sequences are shown in Table 1. PCR products were separated by agarose gel electrophoresis and stained with ethidium bromide. The expression levels of each sample were normalized against GAPDH mRNA expression. The relative mRNA levels of ALP, osteocalcin, and osterix were evaluated by real time PCR using SYBR Green Master kit (Kapa Biosystems, Woburn, MA). Gene specific primer sequences are shown in Table 1. Reactions were performed in triplicate on ABI PRISM 7300 unit (Applied Biosystems). The relative expression levels were calculated using the comparative C_T_ method (ΔΔC_T_) and values were normalized to actin level as an internal control gene. Melting curve analysis was included to assure that only one PCR product was formed.

**Table 1:** Primer sequences for Reverse transcription PCR and real time PCR analysis.

	Primer sequence (5′ - 3′)	
Gene	Forward	Reverse
ALP	GCCCTCTCCAAGACATATA	CCATGATCACGTCGATATCC
Osterix	CTGGGGAAAGGAGGCACAAAGAAG	GGGTTAAGGGGAGCAAAGTCAGAT
Osteopontin	CAGTGATTTGCTTTTGCCTGTTTG	GGTCTCATCAGACTCATCCGAATG
Adipsin	ATGGTATGATGTGCAGAGTGTAG	CACACATCATGTTAATGGTGAC
PPARγ2	GGGTGAAACTCTGGGAGATTCTC	TCAGCAACCATTGGGTCAG
GSK3β	CTTGGACAAAGGTCTTCCGGCC	GTTGGCAGGCGGTGAAGCAG
β-catenin	TGCTGAAGGTGCTGTCTGTC	CTGCTTAGTCGCTGCATCTG
GAPDH	CCCTTCATTGACCTCAACTAC	CCAAAGTTGTCATGGATGACC
* ALP	GCTGATCATTCCCACGTTTT	CTGGGCCTGGTAGTTGTTGT
* Osterix	TGAGGAAGAAGCCCATTCAC	ACTTCTTCTCCCGGGTGTG
* Osteopontin	CGATGTCCCCAACGGCCGAG	TGCTCAGAAGCTGGGCAACAGG
* β-Actin	GCTTCTTTGCAGCTCCTTCGT	ATCGTCATCCATGGCGAACT

* Primer sequences for real time PCR analysis.

### Transfection and luciferase reporter assay

HEK293T cells were cotransfected with the experimental TCF/LEF reporter constructs (TOPFLASH or FOPFLASH) and other expression plasmids, as specified in the figure legands. Luciferase activity was measured by the luciferase assay system (Promega) and normalized to the activity of the control (pRenilla). The data were obtained from three independent transfections and presented as the -fold induction in luciferase activity (mean ± S.D.) relative to the control.

### Generation of transgenic mice

A cDNA encoding HA-tagged human GSK3β mutants was cloned into the expression vector pCAGGS, which carries the cytomegalovirus (CMV) enhancer and chicken β-actin promoter (CAG) and the polyadenylate (polyA) DNA fragments. For generating transgenic mice, we used the standard pronuclear injection method with C57BL/6 mice (The Jackson Laboratory). Genomic DNA isolated from the tail was analyzed by polymerase chain reaction (PCR) using the specific primers (AG-F, 5′-ATGGTAATCGTGCGAGAGGG; AG-R, 5′-CACTGTTGTCACCTTGCTGC; GP-F, 5′-GAGACCGTGGACAGACCAATA; GP-R, 5′-AGCCAGAAGTCAGATGCTCAAG) to detect the transgene.

### Statistical analysis

The data are presented as mean ± S.D. The student's *t*-test was used for statistical analysis. *P*<0.05 was considered statistically significant.

More information is available in Methods S1.

## Results

### Overexpression of GSK3β promotes osteogenic differentiation in ADSCs

**Figure 1:**
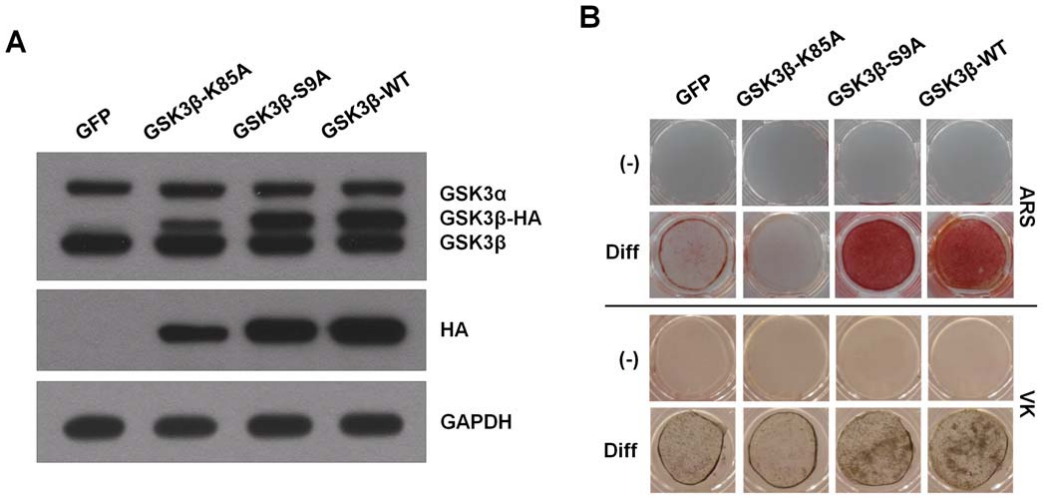
GSK3β activity regulates osteoblast differentiation in ADSCs. (A) ADSCs were infected with either the retrovirus expressing GFP, catalytically inactive GSK3β (GSK3β-K85A), constitutively active GSK3β (GSK3β-S9A), or wild-type GSK3β (GSK3β-WT). After 48 hours, cells were harvested for immunoblot analysis for GSK3α/β expression using antibodies specific for GSK3α/β. It should be noted that due to the increased size of the HA-tagged GSK3β, the protein migrates at a higher molecular mass than that of endogenous GSK3β. The proper expression of transiently transfected HA-tagged GSK3β proteins was further confirmed by immunoblotting with anti-HA antibody. GAPDH served as a loading control. (B) Retroviral infected cells were cultured in osteogenic differentiation medium for 2 weeks. Enhanced matrix mineralization was visualized by Alizarin red S (upper, ARS) and von Kossa (lower, VK) staining. These results are representative of at least three independent experiments.

To determine the role of GSK3β in osteogenic differentiation, murine ADSCs were isolated from mouse inguinal and lateral abdominal fat. We defined ADSCs as those cells that express the cell surface receptor molecule CD44 (hyaluronate) and Sca-1, as well as CD105 (endoglin) but not the hematopoietic markers CD45 and CD11b, and the endothelial markers CD31 (Figure S1A). Further, we showed that ADSCs could differentiate into osteoblasts and adipocytes (Figure S1B). These results showed that ADSCs have characteristics of MSCs and multilineage potential. ADSCs were infected with retroviruses expressing wild-type GSK3β or its two mutants, catalytically inactive GSK3β (GSK3β-K85A) or constitutively active GSK3β (GSK3β-S9A). The GSK3β-transduced cells showed increased GSK3β protein levels (Figure 1A). After differentiation, calcium content was visualized by Alizarin Red S (ARS) and von Kossa (VK) staining. ADSCs infected with GSK3β-K85A showed a marked decrease in osteogenic capacity as indicated by both ARS and VK staining (Figure 1B). Conversely, overexpression of wild-type GSK3β or GSK3β-S9A mutant in ADSCs increased osteogenic capacity of ADSCs in vitro (Figure 1B). These results indicate that forced expression of GSK3β promotes osteogenic differentiation of primary murine ADSCs.

### Silencing of GSK3β by RNA interference blocks osteogenic differentiation in ADSCs

**Figure 2:**
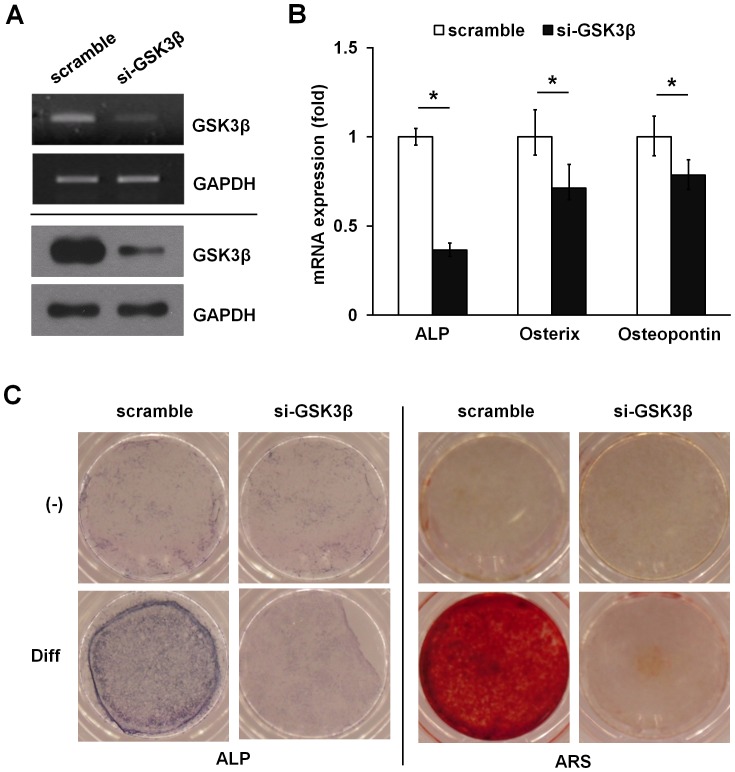
Knockdown of endogenous GSK3β by siRNA inhibits osteoblast differentiation in ADSCs. (A–C) ADSCs were transiently transfected with 10 nM GSK3β siRNA or a nonrelevant siRNA (scramble). After 48 hours, GSK3β silencing was determined by both mRNA (upper, RT-PCR analysis) and protein (lower, immunoblot analysis) levels, respectively. GAPDH is provided as a loading control (A). GSK3β silencing reduced ALP, osterix, and osteopontin mRNA expression determined by real time PCR analysis (B). Silencing GSK3β abolished ALP activity and matrix mineralization as determined by ARS (C). Data represent mean ± S.D. and are representative of at least 3 experiments. *p≤0.01, significantly different from scramble siRNA transfected group.

To establish the role of GSK3ß in osteogenic differentiation of ADSCs, we analyzed the effect of GSK3β on osteogenesis using a small interfering RNA (siRNA)-mediated knocked down experiment. Silencing of GSK3β expression decreased GSK3β mRNA by 60–70% and GSK3β protein level by 70–80%, whereas a control siRNA had no effect (Figure 2A). In contrast to the control siRNA, the GSK3β siRNA decreased ALP, osterix, and osteopontin mRNA levels, supporting a role for GSK3β in osteoblast gene induction in ADSCs (Figure 2B). Consistent with this effect, GSK3β silencing abolished ALP activity and blocked the osteogenic capacity in ADSCs (Figure 2C). Taken together, these data suggest that GSK3β plays a critical role in osteogenic differentiation of ADSCs.

### Effect of GSK3 inhibitors on osteogenic differentiation in ADSCs

**Figure 3:**
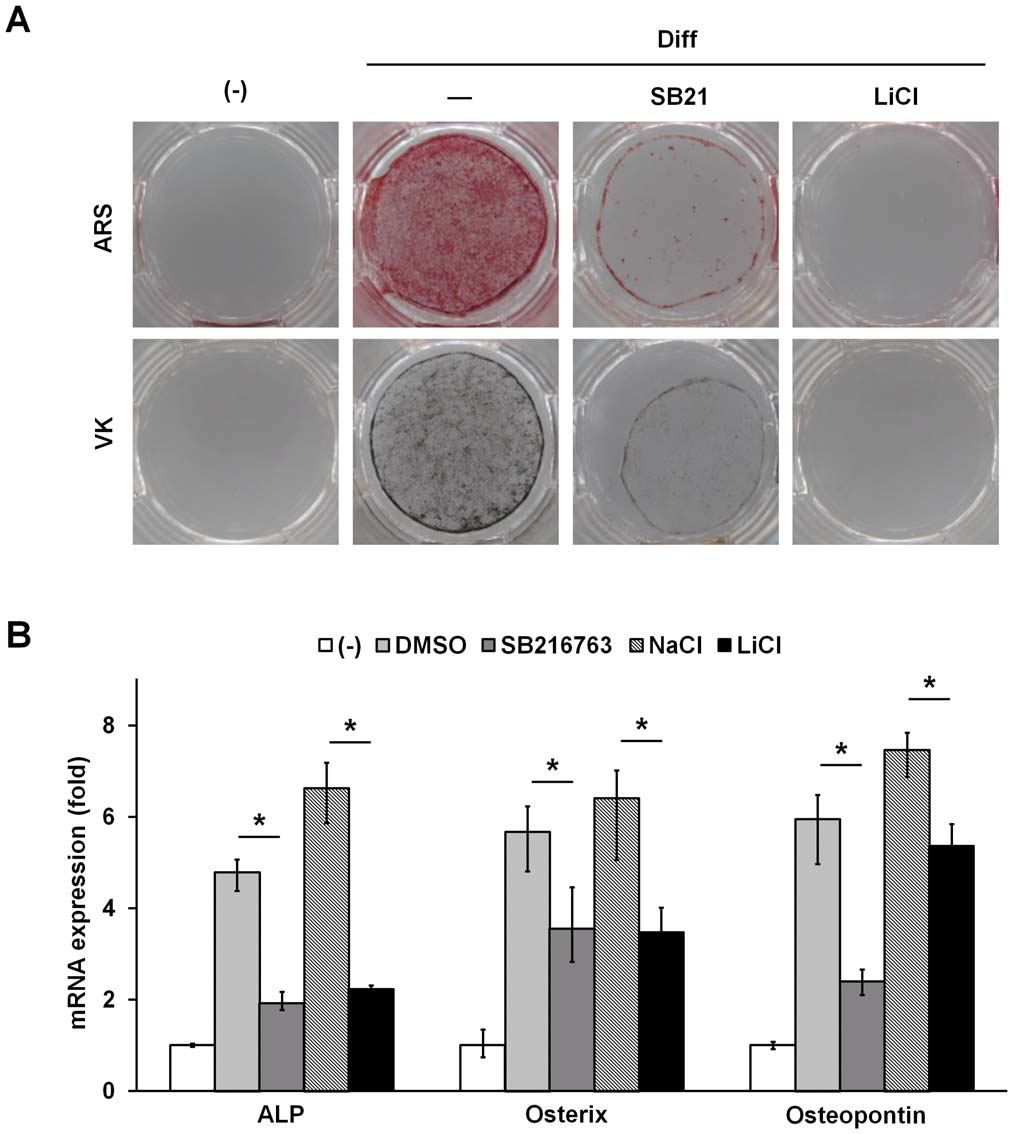
GSK3 inhibitors inhibit osteoblast differentiation in ADSCs. (A) ADSCs were grown to confluence and then cultured in osteogenic differentiation medium with and without 5 μΜ SB216763 (SB21) or 20 mM lithium chloride (LiCl) for 12 days. Subsequently, cells were subjected to ARS (upper) and VK (lower, VK) staining. (B) Total RNA was isolated from cells as in (A) and analyzed for ALP, osterix, and osteopontin expression by real time PCR. DMSO and NaCl were used as the vehicle control. Data represent mean ± S.D. and are representative of at least 3 experiments. *p≤0.01, significantly different from without GSK3β inhibitors group.

To further confirm the role of GSK3β in the differentiation of ADSCs into osteoblasts, we investigated the effects of pharmacological inhibition of GSK3 on osteoblast formation under osteoinductive media. Consistently, incubation of GSK3 inhibitors SB216763 or lithium chloride (LiCl), with ADSCs blocked osteogenic capacity as indicated by both ARS and VK staining (Figure 3A). As shown in Figure 3B, the GSK3 inhibitors decreased ALP, osterix, and osteopontin expression in ADSCs. Collectively, these data point to the importance GSK3β in the osteogenic differentiation process.

### Transgenic expression of GSK3β promotes osteogenic differentiation in ADSCs

**Figure 4:**
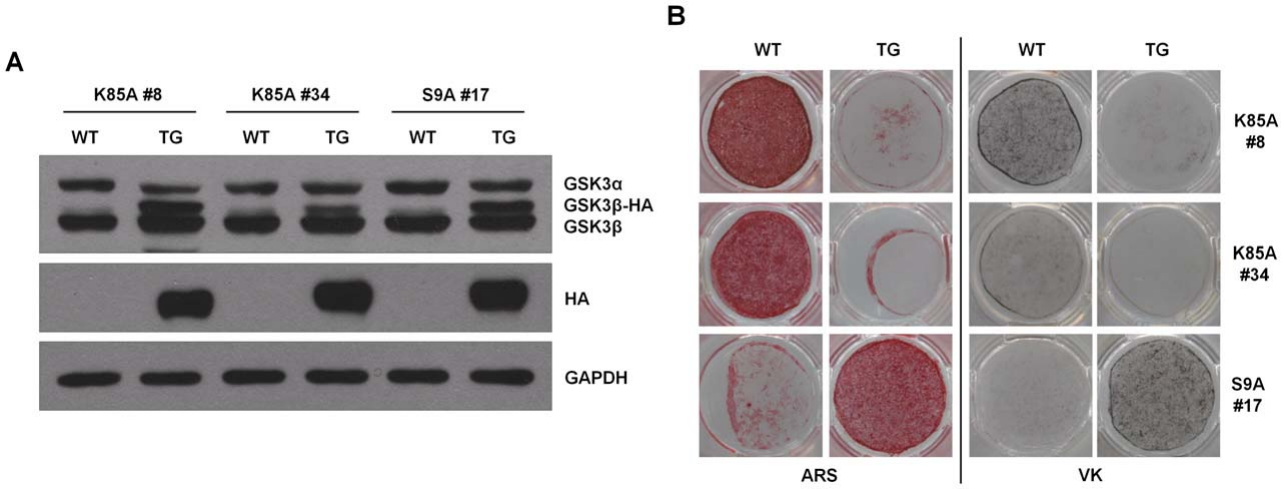
GSK3β regulates osteoblast differentiation in ADSCs of transgenic mice expressing GSK3β mutants. (A) Immunoblot analysis to detect transgene expression in ADSCs purified from the adipose tissues of C57BL/6 mice expressing a transgene (TG) encoding HA-tagged GSK3β or wild-type littermates. As in Fig. 1A, HA-tagged GSK3β protein migrates at a higher molecular mass than that of endogenous GSK3β. The proper expression of HA-tagged GSK3β proteins was further confirmed by immunoblotting with anti-HA antibody. GAPDH served as a loading control. Two independent lines of catalytically inactive GSK3β (K85A #8, K85A #34) and one line of constitutively active GSK3β (S9A #17) were tested for transgene expression. (B) ADSCs purified from GSK3β-K85A TG mice were typically cultured in osteogenic differentiation medium for up to 3 weeks. In contrast, since ADSCs from GSK3β-S9A TG mice respond more rapidly to osteogenic medium, the cells were typically cultured only for 2 weeks. Matrix mineralization was determined by ARS (left) and VK (right) staining. These results are representative of at least three independent experiments.

To clarify the role of GSK3β in vivo, we generated transgenic (Tg) mice expressing GSK3β-S9A or GSK3β-K85A under control of the ß-actin gene promoter. Human GSK3β-S9A or GSK3β-K85A cDNA tagged hemagglutinin (HA) was fused with a 1.3-kb sequence located upstream of the ß-actin gene (Figure S2A). GSK3β Tg mice were generated by injecting a linear DNA fragment into the pronuclei of fertilized eggs of C57BL/6 mice. Three lines (#8 & #34 for GSK3β-K85A; #17 for GSK3β-S9A) of GSK3β Tg mice were selected by PCR of the tail DNA using transgene-specific primers (Figure S2B). We confirmed that HA-tagged GSK3β was expressed in ADSCs of the GSK3β Tg mice (Figure 4A). We first examined the bone phenotype of transgenic mice expressing GSK3β-K85A or GSK3β-S9A. Notably, there was no significant difference in the trabecular bone volume or the bone mineral density between wild-type and transgenic mice (Figure S3). Next, we investigated the functional consequence of GSK3β overexpression in mice on proliferation of ADSCs. We did not observe any significant change of ADSCs proliferation by expression of GSK3β-K85A or GSK3β-S9A mutants (Figure S4). However, consistent with the previous findings, osteogenic capacity of ADSCs from Tg mice expressing GSK3β-K85A mutant was markedly lower than that of wild-type littermates. Conversely, expression of GSK3β-S9A mutant in Tg mice increased osteogenic differentiation in ADSCs (Figure 4B). These results indicate that forced expression of GSK3β promotes the ex vivo osteogenic differentiation.

### Effects of GSK3β on intracellular protein levels of β-catenin

**Figure 5:**
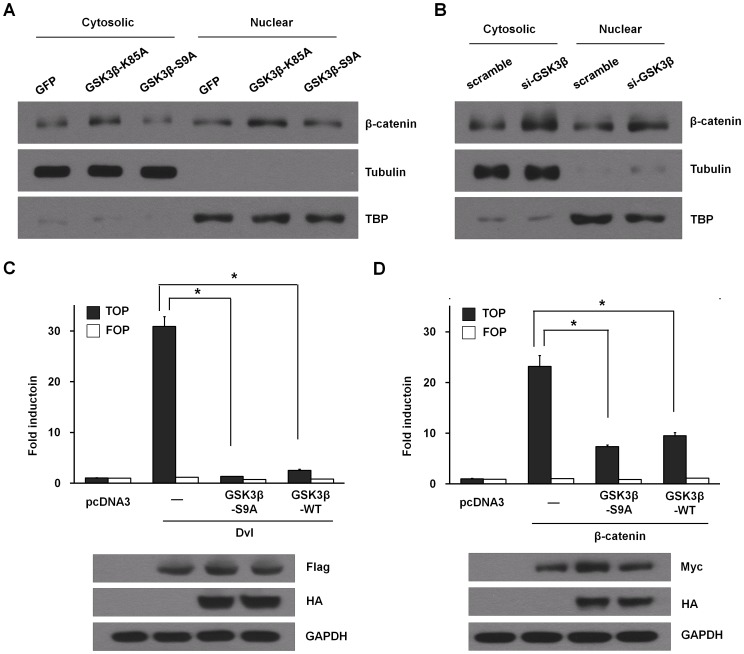
GSK3β activity regulates β-catenin level in ADSCs. (A) ADSCs were infected with a retrovirus expressing GFP, GSK3β-K85A, or GSK3β-S9A for 24 hours and infected cells were selected with 2 μg/ml puromycin. After 48 hours, the cells were fractionated into nuclear and cytosolic fractions and immunoblotted using β-catenin antibody. The purity of fractions was confirmed with TATA-binding protein (TBP) and tubulin antibodies, respectively. (B) ADSCs were transiently transfected with 10 nM GSK3β siRNA or a nonrelevant siRNA (scramble). After 48 hours, cells were processed and analyzed as in (A). (C, D) HEK293T cells were transfected with a wild-type TCF/LEF reporter (TOPFLASH) and a mutant inactive form (FOPFLASH), together with the indicated constructs and promoter activity, was measured by luciferase assay 24 hours later. The luciferase activities were normalized for pRenilla, the control reporter, activity. Overexpression of Flag-tagged Dvl (C) or Myc-tagged β-catenin (D) together with HA-tagged GSK3β-S9A or WT was detected by immunoblotting. Data represent mean ± S.D. and are representative of at least 3 experiments. *p≤0.01, significantly different from mouse Dvl-Flag transfected group or mouse β-catenin-myc transfected group.

Inhibition of GSK3β decreases phosphorylation of β-catenin, preventing its degradation by the proteasome [26]. Stabilized β-catenin acts on the nucleus by activating T cell factor/lymphoid enhancing factor (TCF/Lef)-mediated transcription of target genes that elicit a variety of effects, including the induction of differentiation. We examined whether inhibition of GSK3β would mimic Wnt signaling through direct stabilization of β-catenin, therefore also resulting in enhancement of Wnt signaling [26]–[28]. Both retroviral transfer of GSK3β-K85A mutant and silencing of GSK3β in ADSCs under osteogenic conditions resulted in increased levels of β-catenin in the cytoplasm and the nucleus, compared to that of control (Figure 5A and B). Consistently, pharmacological inhibition of GSK3 in ADSCs increased protein levels of β-catenin (Figure S5). Next, we quantified the ability of GSK3β to regulate canonical Wnt signaling by measuring transcriptional activation of β-catenin-inducible TCF/Lef luciferase reporter. The results showed that overexpression of Dishevelled (Dvl) or β-catenin resulted in a marked increase in the TCF/Lef reporter activity (Figure 5C and D). Wild-type GSK3β or GSK3β-S9A mutant clearly inhibited the activation of the reporter by both Dvl and β-catenin. These results suggest that the canonical Wnt axis might be inhibited in ADSCs by overexpression of GSK3β.

### Silencing of β-catenin promotes osteogenic differentiation in ADSCs

**Figure 6:**
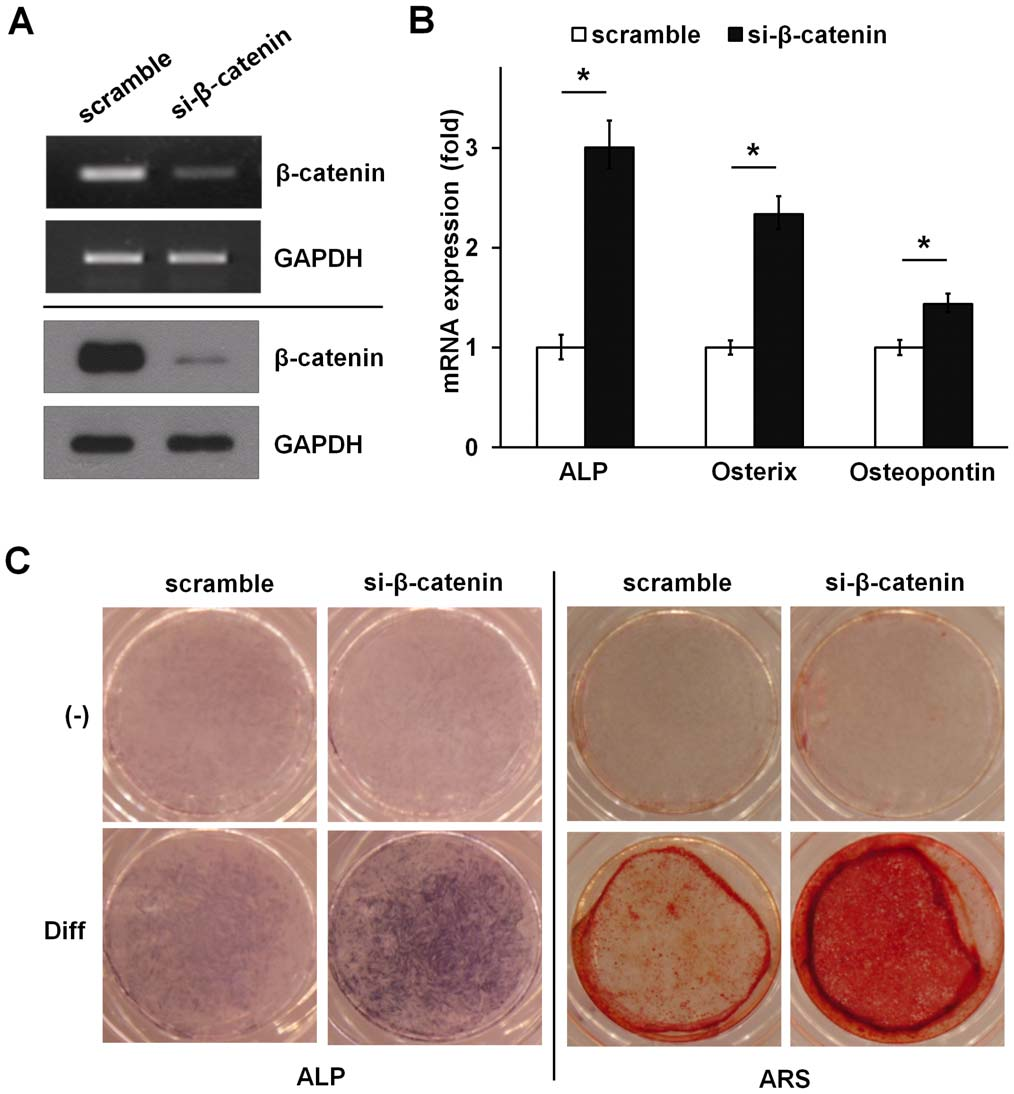
Knockdown of endogenous β-catenin enhances osteoblast differentiation of ADSCs. (A–C) ADSCs were transiently transfected with 10 nM β-catenin siRNA or a nonrelevant siRNA (scramble). After 48 hours, β-catenin silencing was determined by both mRNA (upper, RT-PCR analysis) and protein (lower, immunoblot analysis) levels, respectively. GAPDH is provided as a loading control (A). β-catenin silencing increased ALP, osterix, and osteopontin mRNA expression determined by real time PCR analysis (B). Silencing β-catenin enhanced ALP activity. Matrix mineralization as determined by ARS (C). Data represent mean ± S.D. and are representative of at least 3 experiments. *p≤0.01, significantly different from scramble siRNA transfected group.

To investigate the role of β-catenin in osteogenic differentiation of ADSCs, we determined whether siRNA-mediated silencing of β-catenin regulates expression of osteoblast marker genes in ADSCs. Silencing of β-catenin decreased β-catenin mRNA by 60% and β-catenin protein level by 90%, whereas a control siRNA had no effect (Figure 6A). In contrast to the control siRNA, the β-catenin siRNA increased mRNA levels of ALP, osterix and osteopontin (Figure 6B). Consistent with this effect, β-catenin silencing increased osteogenic capacity as indicated by both ALP and ARS staining (Figure 6C). These results indicate that β-catenin negatively regulates osteogenic differentiation in ADSCs.

## Discussion

The characterization of regulatory mechanisms that direct osteogenic differentiation of ADSCs is of prime interest for developing therapeutic strategies to enhance bone formation and regeneration [1]. Canonical Wnt/β-catenin signaling has been suggested to have a critical role in the osteogenic processes in BMSCs. In the activation of the canonical Wnt pathway, inhibition of GSK3β results in dephosphorylation of β-catenin leading to its nuclear accumulation [18], [31]. In this study, we establish the positive role of GSK3β in ADSCs differentiation. We report that ADSCs, when exposed to an uncontrolled rise of the β-catenin level due to loss-of-function of GSK3β activity, by either pharmacological inhibition or by gene silencing, lose their differentiation capacity to the osteoblasts. Notably, forced expression of GSK3β-S9A, the constitutively active GSK3β mutant by either retroviral transfer or by a transgenic approach increased osteogenic capacity. Consistent with this finding, specific β-catenin silencing increased osteoblast marker gene expression and prompted the in vitro osteogenic capacity of ADSCs.

The effect of Wnt pathway on osteogenic differentiation remains more controversial. Early studies have yielded a wealth of information describing that activation of the Wnt/β-catenin signaling pathway promotes osteogenic differentiation [32]–[35]. Consistent with these previous findings, we observed that GSK3β inhibition by either overexpression of GSK3β-K85A or by gene silencing positively regulates osteogenesis of BMSCs (Figure S6). Paradoxically, and in agreement with our results, a number of articles have recently claimed that Wnt/β-catenin signaling inhibits osteogenic differentiation and mineralization of MSCs in vitro [36]–[43]. However, the mechanism seems more complex. For example, a recent study indicated that the Wnt/β-catenin pathway suppresses osteogenic differentiation of human adipose-derived MSCs [39]–[41]. In this case, inhibition of MSC osteogenesis via the Wnt/β-catenin pathway is associated with decreased expression of osteoblastic transcription factors and inhibition of mitogen-activated protein kinases activation, which are involved in osteogenic differentiation [44]. Boer et al., [37] have shown that there was a balance involved in MSC differentiation, and canonical Wnts are both osteoinhibitory and osteoinductive depending on the extent of signaling. Boland et al., [38] demonstrated that canonical Wnts, including Wnt3a and Wnt1, inhibit osteogenic differentiation but promote proliferation of MSCs in vitro. More recently, Zaragosi et al., [45] have shown that GSK3 plays a role in maintaining differentiation potential of undifferentiated hADSCs. They showed that ALP activity was inhibited when hADSCs were maintained in osteogenic media supplemented with GSK3 inhibitor, 6-bromoindirubin-3′-oxime (BIO). They suggested that BIO-treated hADSCs had a negative effect of Wnt on osteogenesis, indicating that GSK3β might have a positive role on osteogenesis. Although ADSCs are display similar characteristics as BMSCs in terms of cell surface receptors expression and multilineage potential (Figure S7), the mechanism and pathways that these cells utilize to differentiate into osteoblasts are might be different. More detailed analysis of how the two MSC types differ is necessary to understand the biology of MSCs obtained from different tissues and to delineate their full potential and clinical significance [46].

In light of a series of experiments carried out through this study, our results suggest that the regulation of GSK3β-mediated β-catenin level is critical for osteogenic differentiation of ADSCs. It is possible that high levels of β-catenin negatively regulate the differentiation of ADSCs into an osteoblast precursor. Therefore, an early phase of differentiation process of ADSCs may maintain lower levels of β-catenin. However, in order that the precursors further differentiate into osteoblasts, they may elevate β-catenin levels. In agreement with this hypothesis, we observed differential expression patterns of β-catenin over a period of time during osteogenesis (Figure S8). At the early phase of differentiation under osteoinductive media, protein levels of β-catenin are significantly reduced. In contrast, β-catenin levels increased significantly after 8 days under osteogenic condition, suggesting that β-catenin may act as an inhibitor in the early stages of osteogenesis and as an inducer later on.

In conclusion, here we provide evidence supporting the finding that GSK3β positively regulates osteogenic differentiation of ADSCs. The identified effects of GSK3β were due to regulation of β-catenin levels. Enhanced GSK3β expression had a significant positive effect on osteogenic capacity through downregulation of β-catenin. It might be clear that maintenance of an appropriate physiological level of GSK3β activity is crucial since either too little or too much GSK3β activity can modulate cell fate changes. Our data now suggest another level of regulation in osteogenic differentiation of ADSCs and may help to explain the complexity of cellular responses to GSK3β/β-catenin signaling.

## Supporting Information

Figure S1 **Immunophenotypic characterization and differentiation assays of mouse adipose-derived stromal cells (ADSCs).** (A) ADSCs were stained with antibodies against surface markers or control antibodies and subjected to flow cytometry analysis. Cells were homogenously positive for mesenchymal markers CD44 and CD105; progenitor cells markers Sca-1 but negative for hematopoietic markers CD11b and CD45 and endothelial markers CD31. The respective isotype control is shown as a thick black-line histogram. (B, C) ADSCs were cultured with or without differentiation medium for 1 to 2 weeks to induce cell differentiation. Osteogenesis was detected by Von Kossa (VK) staining and adipogenesis was detected by Oil red O (ORO) staining (Original magnification,×10). Expression of osteoblast and adipocyte marker encoding genes was analysed by RT-PCR. The osteoblast markers, alkaline phosphatase (ALP), osteopontin, and osterix were expressed in differentiated cultures, whereas control undifferentiated cultures were negative for these genes (B). The adipocytic markers, adipsin and PPARγ2 were expressed in differentiated cells (C). Glyceraldehyde 3 phosphate dehydrogenase (GAPDH) is shown as a loading control.
(PDF)

Figure S2 **Generation of transgenic mice expressing GSK3β mutants.** (A) Schematic representation of the transgenic construct. The HA-tagged human GSK3β (K85A, S9A) cDNA was cloned into the expression vector pCAGGS, which carries the cytomegalovirus (CMV) enhancer, the chicken β-actin promoter (CAG) and the polyadenylate (polyA) DNA fragments. (B) Schematic diagram of the GSK3β constructs shown in (A) and arrows indicate a set of primers used for confirming integration of the transgene. Genomic DNA isolated from the tail of transgenic mice (TG) and wild-type littermates (WT) was analyzed by PCR using specific primers for the transgene.
(PDF)

Figure S3 **Three-dimensional microstructural analysis of the femurs of wild- type (WT) and Tg mice (Tg) by microcomputed tomography (μCT).** (A) S9A #17 Tg and WT littermates. (B) K85A #8 Tg and WT littermates. (C) K85A #34 Tg and WT littermates. Histograms represent the three-dimensional trabecular structural parameters in femurs: bone volume fraction (BV/TV), trabecular number (Tb. N), and bone mineral densities (BMD). Data represent means ± SD. *n* = 6. n.s., not significant.
(PDF)

Figure S4 **Effect of GSK3β on the proliferation of ADSCs in transgenic mice expressing GSK3β mutants.** ADSCs were isolated from either GSK3β-K85A (#8 and #34 line) or GSK3β-S9A (#17 line) Tg mice and WT littermates. ADSCs were maintained in medium supplemented with 10% FCS. Cell proliferation was determined by the MTT assay during 7 days of culture.
(PDF)

Figure S5 **GSK3 inhibitors induce β-catenin accumulation in ADSCs.** ADSCs were treated with 5 μΜ SB216763 (SB21) or 20 mM LiCl in osteogenic differentiation medium for the indicated periods of time. Whole cell extracts were immunoblotted with total β-catenin, phospho-Ser^9^-GSK3β antibody and GSK3β antibody. Membranes were reblotted with GAPDH antibody to ensure equal protein loading. These results are representative of at least three independent experiments.
(PDF)

Figure S6 **Effects of GSK3β on osteoblast differentiation in BMSCs.** (A) BMSCs were infected with either the retrovirus expressing GFP, catalytically inactive GSK3β (GSK3β-K85A) or constitutively active GSK3β (GSK3β-S9A). After 48 hours, cells were harvested for immunoblot analysis for GSK3β expression using antibodies specific for GSK3β or HA. GAPDH served as a loading control. (B) Retroviral infected cells were cultured in osteogenic differentiation medium for 2 weeks. Alkaline phosphatase activity and matrix mineralization were visualized by ALP (ALP) and Alizarin red S (ARS) staining. (C) Total RNA was isolated and analyzed for ALP, osterix, and osteopontin expression by real time PCR. Data represent mean ± S.D. *p<0.05, **p<0.001. Significant differences from the GFP infected group are seen. (D) BMSCs were transiently transfected with 10 nM GSK3β siRNA or a control siRNA (scramble). After 48 hours, GSK3β silencing was examined by immunoblot analysis. GAPDH is used as a loading control. Silencing GSK3β enhanced ALP activity and matrix mineralization as determined by ARS staining. Further, GSK3β silencing increased ALP, osterix and osteopontin mRNA expression as determined by real time PCR analysis. Data represent mean ± S.D. *p<0.001 and show significant difference from scramble siRNA transfected group.
(PDF)

Figure S7 **Immunophenotypic characterization and differentiation assays of BMSCs.** (A) BMSCs were stained with antibodies against surface markers or control antibodies and subjected to flow cytometry analysis. Cells were homogeneously positive for mesenchymal markers CD44 and CD105 as well as for progenitor cells markers Sca-1 but negative for hematopoietic markers CD11b and CD45 and endothelial markers CD31. The respective isotype control is shown as a thick black-line in the histogram. (B, C) BMSCs were cultured with or without differentiation medium for 2 weeks to induce cell differentiation. Osteoblastogenesis was detected by Alizarin red S (ARS) staining, and adipogenesis was detected by Oil red O (ORO) staining. Original magnification, X 10.
(PDF)

Figure S8 **Change of β-catenin levels during osteoblast differentiation.** ADSCs were cultured in osteogenic differentiation medium for the indicated periods of time. Whole cell extracts were immunoblotted with total β-catenin. Membranes were reblotted with GAPDH antibody to ensure equal protein loading. These results are representative of at least three independent experiments.
(PDF)

Methods S1 **More information on the Flow cytometric analysis, BMSC isolation, Adipogenic differentiation and oil red O (ORO) staining, and MicroCT analysis.**
(DOC)

## References

[1] Levi B, Longaker MT (2011) Concise review: adipose-derived stromal cells for skeletal regenerative medicine. Stem Cells 29: 576–582. 21305671 10.1002/stem.612 PMC3323288

[2] Zuk PA, Zhu M, Ashjian P, De Ugarte DA, Huang JI, et al (2002) Human adipose tissue is a source of multipotent stem cells. Mol Biol Cell 13: 4279–4295. 12475952 10.1091/mbc.E02-02-0105 PMC138633

[3] Reyes M, Lund T, Lenvik T, Aguiar D, Koodie L, et al (2001) Purification and ex vivo expansion of postnatal human marrow mesodermal progenitor cells. Blood 98: 2615–2625. 11675329 10.1182/blood.v98.9.2615

[4] Majumdar MK, Thiede MA, Haynesworth SE, Bruder SP, Gerson SL (2000) Human marrow-derived mesenchymal stem cells (MSCs) express hematopoietic cytokines and support long-term hematopoiesis when differentiated toward stromal and osteogenic lineages. J Hematother Stem Cell Res 9: 841–848. 11177595 10.1089/152581600750062264

[5] Pereira RF, Halford KW, O'Hara MD, Leeper DB, Sokolov BP, et al (1995) Cultured adherent cells from marrow can serve as long-lasting precursor cells for bone, cartilage, and lung in irradiated mice. Proc Natl Acad Sci U S A 92: 4857–4861. 7761413 10.1073/pnas.92.11.4857 PMC41806

[6] Pittenger MF, Mackay AM, Beck SC, Jaiswal RK, Douglas R, et al (1999) Multilineage potential of adult human mesenchymal stem cells. Science 284: 143–147. 10102814 10.1126/science.284.5411.143

[7] Zuk PA, Zhu M, Ashjian P, De Ugarte DA, Huang JI, et al (2002) Human adipose tissue is a source of multipotent stem cells. Mol Biol Cell 13: 4279–4295. 12475952 10.1091/mbc.E02-02-0105 PMC138633

[8] Awad HA, Halvorsen YD, Gimble JM, Guilak F (2003) Effects of transforming growth factor beta1 and dexamethasone on the growth and chondrogenic differentiation of adipose-derived stromal cells. Tissue Eng 9: 1301–1312. 14670117 10.1089/10763270360728215

[9] Ogawa R, Mizuno H, Watanabe A, Migita M, Hyakusoku H, et al (2004) Adipogenic differentiation by adipose-derived stem cells harvested from GFP transgenic mice-including relationship of sex differences. Biochem Biophys Res Commun 319: 511–517. 15178436 10.1016/j.bbrc.2004.05.021

[10] Rich JT, Rosova I, Nolta JA, Myckatyn TM, Sandell LJ, et al (2008) Upregulation of Runx2 and Osterix during in vitro chondrogenesis of human adipose-derived stromal cells. Biochem Biophys Res Commun 372: 230–235. 18482578 10.1016/j.bbrc.2008.05.022 PMC2548292

[11] Bianco P, Robey PG (2001) Stem cells in tissue engineering. Nature 414: 118–121. 11689957 10.1038/35102181

[12] Prockop DJ, Gregory CA, Spees JL (2003) One strategy for cell and gene therapy: harnessing the power of adult stem cells to repair tissues. Proc Natl Acad Sci U S A 100 Suppl 111917–11923. 13679583 10.1073/pnas.1834138100 PMC304107

[13] Petite H, Viateau V, Bensaid W, Meunier A, de Pollak C, et al (2000) Tissue-engineered bone regeneration. Nat Biotechnol 18: 959–963. 10973216 10.1038/79449

[14] Hamidouche Z, Fromigue O, Ringe J, Haupl T, Vaudin P, et al (2009) Priming integrin alpha5 promotes human mesenchymal stromal cell osteoblast differentiation and osteogenesis. Proc Natl Acad Sci U S A 106: 18587–18591. 19843692 10.1073/pnas.0812334106 PMC2773973

[15] Bandyopadhyay A, Tsuji K, Cox K, Harfe BD, Rosen V, et al (2006) Genetic analysis of the roles of BMP2, BMP4, and BMP7 in limb patterning and skeletogenesis. PLoS Genet 2: e216. 17194222 10.1371/journal.pgen.0020216 PMC1713256

[16] Yoon BS, Ovchinnikov DA, Yoshii I, Mishina Y, Behringer RR, et al (2005) Bmpr1a and Bmpr1b have overlapping functions and are essential for chondrogenesis in vivo. Proc Natl Acad Sci U S A 102: 5062–5067. 15781876 10.1073/pnas.0500031102 PMC555995

[17] Day TF, Guo X, Garrett-Beal L, Yang Y (2005) Wnt/beta-catenin signaling in mesenchymal progenitors controls osteoblast and chondrocyte differentiation during vertebrate skeletogenesis. Dev Cell 8: 739–750. 15866164 10.1016/j.devcel.2005.03.016

[18] Hartmann C (2006) A Wnt canon orchestrating osteoblastogenesis. Trends Cell Biol 16: 151–158. 16466918 10.1016/j.tcb.2006.01.001

[19] Davis LA, Zur Nieden NI (2008) Mesodermal fate decisions of a stem cell: the Wnt switch. Cell Mol Life Sci 65: 2658–2674. 18528633 10.1007/s00018-008-8042-1 PMC2778684

[20] Engin F, Yao Z, Yang T, Zhou G, Bertin T, et al (2008) Dimorphic effects of Notch signaling in bone homeostasis. Nat Med 14: 299–305. 18297084 10.1038/nm1712 PMC2671578

[21] Tsutsumi S, Shimazu A, Miyazaki K, Pan H, Koike C, et al (2001) Retention of multilineage differentiation potential of mesenchymal cells during proliferation in response to FGF. Biochem Biophys Res Commun 288: 413–419. 11606058 10.1006/bbrc.2001.5777

[22] Ito T, Sawada R, Fujiwara Y, Tsuchiya T (2008) FGF-2 increases osteogenic and chondrogenic differentiation potentials of human mesenchymal stem cells by inactivation of TGF-beta signaling. Cytotechnology 56: 1–7. 19002835 10.1007/s10616-007-9092-1 PMC2151969

[23] St-Jacques B, Hammerschmidt M, McMahon AP (1999) Indian hedgehog signaling regulates proliferation and differentiation of chondrocytes and is essential for bone formation. Genes Dev 13: 2072–2086. 10465785 10.1101/gad.13.16.2072 PMC316949

[24] Mak KK, Chen MH, Day TF, Chuang PT, Yang Y (2006) Wnt/beta-catenin signaling interacts differentially with Ihh signaling in controlling endochondral bone and synovial joint formation. Development 133: 3695–3707. 16936073 10.1242/dev.02546

[25] Lin GL, Hankenson KD (2011) Integration of BMP, Wnt, and notch signaling pathways in osteoblast differentiation. J Cell Biochem 112: 3491–3501. 21793042 10.1002/jcb.23287 PMC3202082

[26] Cohen P, Frame S (2001) The renaissance of GSK3. Nat Rev Mol Cell Biol 2: 769–776. 11584304 10.1038/35096075

[27] Doble BW, Woodgett JR (2003) GSK-3: tricks of the trade for a multi-tasking kinase. J Cell Sci 116: 1175–1186. 12615961 10.1242/jcs.00384 PMC3006448

[28] Wu D, Pan W (2010) GSK3: a multifaceted kinase in Wnt signaling. Trends Biochem Sci 35: 161–168. 19884009 10.1016/j.tibs.2009.10.002 PMC2834833

[29] Bunnell BA, Flaat M, Gagliardi C, Patel B, Ripoll C (2008) Adipose-derived stem cells: isolation, expansion and differentiation. Methods 45: 115–120. 18593609 10.1016/j.ymeth.2008.03.006 PMC3668445

[30] Jang HD, Shin JH, Park DR, Hong JH, Yoon K, et al (2011) Inactivation of glycogen synthase kinase-3beta is required for osteoclast differentiation. J Biol Chem 286: 39043–39050. 21949120 10.1074/jbc.M111.256768 PMC3234729

[31] Krishnan V, Bryant HU, Macdougald OA (2006) Regulation of bone mass by Wnt signaling. J Clin Invest 116: 1202–1209. 16670761 10.1172/JCI28551 PMC1451219

[32] Krause U, Harris S, Green A, Ylostalo J, Zeitouni S, et al (2010) Pharmaceutical modulation of canonical Wnt signaling in multipotent stromal cells for improved osteoinductive therapy. Proc Natl Acad Sci U S A 107: 4147–4152. 20150512 10.1073/pnas.0914360107 PMC2840116

[33] Bain G, Muller T, Wang X, Papkoff J (2003) Activated beta-catenin induces osteoblast differentiation of C3H10T1/2 cells and participates in BMP2 mediated signal transduction. Biochem Biophys Res Commun 301: 84–91. 12535644 10.1016/s0006-291x(02)02951-0

[34] Rawadi G, Vayssiere B, Dunn F, Baron R, Roman-Roman S (2003) BMP-2 controls alkaline phosphatase expression and osteoblast mineralization by a Wnt autocrine loop. J Bone Miner Res 18: 1842–1853. 14584895 10.1359/jbmr.2003.18.10.1842

[35] Gregory CA, Green A, Lee N, Rao A, Gunn W (2006) The promise of canonical Wnt signaling modulators in enhancing bone repair. Drug News Perspect 19: 445–452. 17160144 10.1358/dnp.19.8.1043960

[36] Liu N, Shi S, Deng M, Tang L, Zhang G, et al (2011) High levels of beta-catenin signaling reduce osteogenic differentiation of stem cells in inflammatory microenvironments through inhibition of the noncanonical Wnt pathway. J Bone Miner Res 26: 2082–2095. 21638320 10.1002/jbmr.440

[37] De Boer J, Wang HJ, Van Blitterswijk C (2004) Effects of Wnt signaling on proliferation and differentiation of human mesenchymal stem cells. Tissue Eng 10: 393–401. 15165456 10.1089/107632704323061753

[38] Boland GM, Perkins G, Hall DJ, Tuan RS (2004) Wnt 3a promotes proliferation and suppresses osteogenic differentiation of adult human mesenchymal stem cells. J Cell Biochem 93: 1210–1230. 15486964 10.1002/jcb.20284

[39] Cho HH, Kim YJ, Kim SJ, Kim JH, Bae YC, et al (2006) Endogenous Wnt signaling promotes proliferation and suppresses osteogenic differentiation in human adipose derived stromal cells. Tissue Eng 12: 111–121. 16499448 10.1089/ten.2006.12.111

[40] van der Horst G, van der Werf SM, Farih-Sips H, van Bezooijen RL, Lowik CW, et al (2005) Downregulation of Wnt signaling by increased expression of Dickkopf-1 and -2 is a prerequisite for late-stage osteoblast differentiation of KS483 cells. J Bone Miner Res 20: 1867–1877. 16160745 10.1359/JBMR.050614

[41] Jian H, Shen X, Liu I, Semenov M, He X, et al (2006) Smad3-dependent nuclear translocation of beta-catenin is required for TGF-beta1-induced proliferation of bone marrow-derived adult human mesenchymal stem cells. Genes Dev 20: 666–674. 16543220 10.1101/gad.1388806 PMC1413283

[42] Gregory CA, Singh H, Perry AS, Prockop DJ (2003) The Wnt signaling inhibitor dickkopf-1 is required for reentry into the cell cycle of human adult stem cells from bone marrow. J Biol Chem 278: 28067–28078. 12740383 10.1074/jbc.M300373200

[43] Li X, Liu P, Liu W, Maye P, Zhang J, et al (2005) Dkk2 has a role in terminal osteoblast differentiation and mineralized matrix formation. Nat Genet 37: 945–952. 16056226 10.1038/ng1614

[44] Liu G, Vijayakumar S, Grumolato L, Arroyave R, Qiao H, et al (2009) Canonical Wnts function as potent regulators of osteogenesis by human mesenchymal stem cells. J Cell Biol 185: 67–75. 19349579 10.1083/jcb.200810137 PMC2700509

[45] Zaragosi LE, Wdziekonki B, Fontaine C, Villageois P, Peraldi P, et al (2008) Effects of GSK3 inhibitors on in vitro expansion and differentiation of human adipose-derived stem cells into adipocytes. BMC Cell Biol 9: 11–20. 18271953 10.1186/1471-2121-9-11 PMC2257931

[46] Rebelatto CK, Aguiar AM, Moretao MP, Senegalia AC, Hansen P, et al (2008) Dissimilar differentiation of mesenchymal stem cells from bone marrow, umbilical cord blood, and adipose tissue. Exp Biol Med 233: 901–913. 10.3181/0712-RM-356 18445775

