# Supplementary figures and images for: Glycogen Synthase Kinase 3β Promotes Osteogenic Differentiation of Murine Adipose-Derived Stromal Cells
Source: PLoS One. 2013 Jan 16;8(1):e54551. doi: 10.1371/journal.pone.0054551 (PMC3546989; doi:10.1371/journal.pone.0054551)

**A**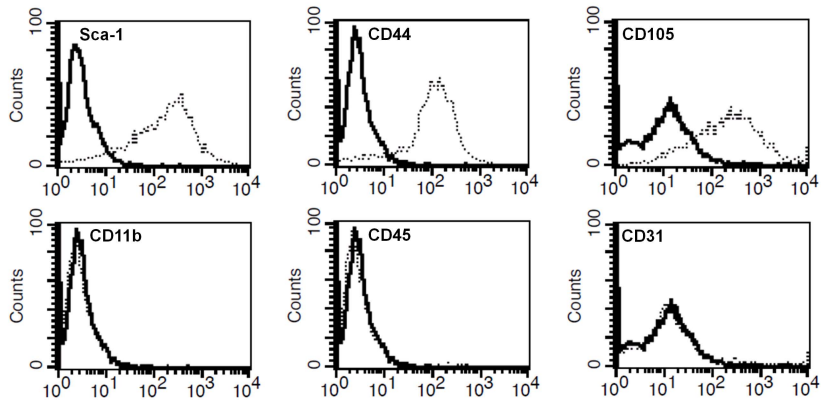**B**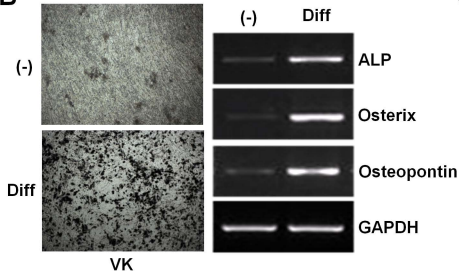**C**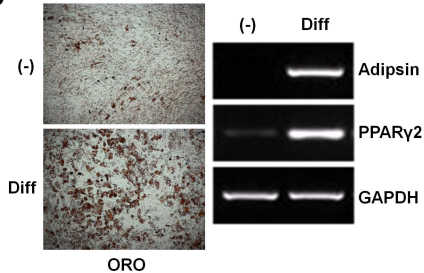

Supplement: Figure S1 — Immunophenotypic characterization and differentiation assays of mouse adipose-derived stromal cells (ADSCs). (A) ADSCs were stained with antibodies against surface markers or control antibodies and subjected to flow cytometry analysis. Cells were homogenously positive for mesenchymal markers CD44 and CD105; progenitor cells markers Sca-1 but negative for hematopoietic markers CD11b and CD45 and endothelial markers CD31. The respective isotype control is shown as a thick black-line histogram. (B, C) ADSCs were cultured with or without differentiation medium for 1 to 2 weeks to induce cell differentiation. Osteogenesis was detected by Von Kossa (VK) staining and adipogenesis was detected by Oil red O (ORO) staining (Original magnification,×10). Expression of osteoblast and adipocyte marker encoding genes was analysed by RT-PCR. The osteoblast markers, alkaline phosphatase (ALP), osteopontin, and osterix were expressed in differentiated cultures, whereas control undifferentiated cultures were negative for these genes (B). The adipocytic markers, adipsin and PPARγ2 were expressed in differentiated cells (C). Glyceraldehyde 3 phosphate dehydrogenase (GAPDH) is shown as a loading control. (PDF) [file pone.0054551.s001.pdf]

**A**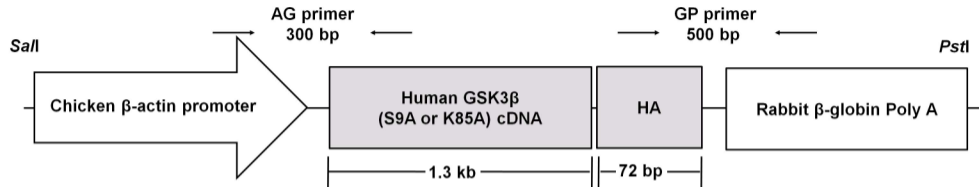**B**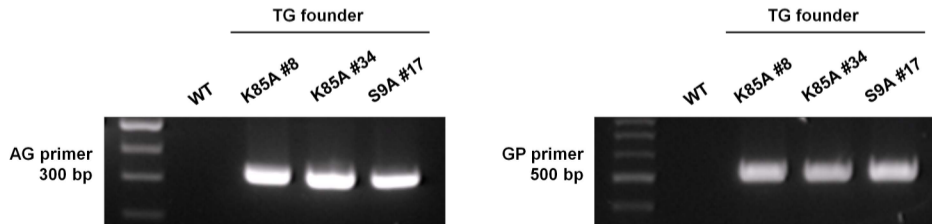

Supplement: Figure S2 — Generation of transgenic mice expressing GSK3β mutants. (A) Schematic representation of the transgenic construct. The HA-tagged human GSK3β (K85A, S9A) cDNA was cloned into the expression vector pCAGGS, which carries the cytomegalovirus (CMV) enhancer, the chicken β-actin promoter (CAG) and the polyadenylate (polyA) DNA fragments. (B) Schematic diagram of the GSK3β constructs shown in (A) and arrows indicate a set of primers used for confirming integration of the transgene. Genomic DNA isolated from the tail of transgenic mice (TG) and wild-type littermates (WT) was analyzed by PCR using specific primers for the transgene. (PDF) [file pone.0054551.s002.pdf]

**A**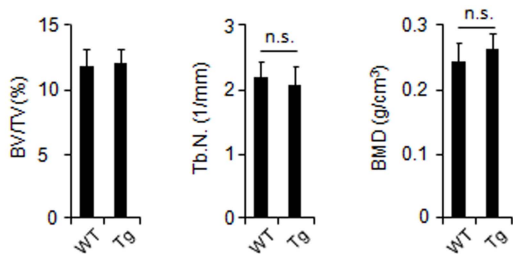**B**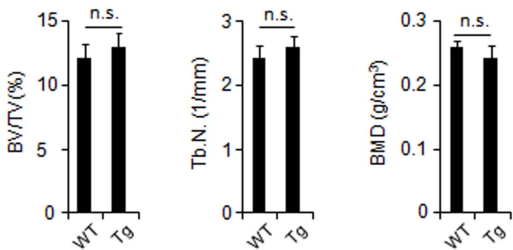**C**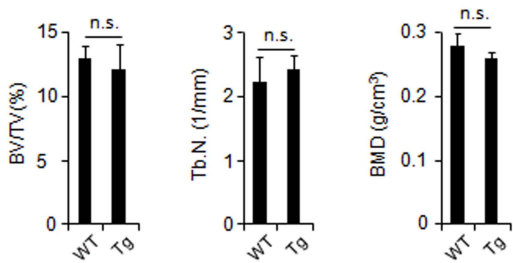

Supplement: Figure S3 — Three-dimensional microstructural analysis of the femurs of wild- type (WT) and Tg mice (Tg) by microcomputed tomography (μCT). (A) S9A #17 Tg and WT littermates. (B) K85A #8 Tg and WT littermates. (C) K85A #34 Tg and WT littermates. Histograms represent the three-dimensional trabecular structural parameters in femurs: bone volume fraction (BV/TV), trabecular number (Tb. N), and bone mineral densities (BMD). Data represent means ± SD. n = 6. n.s., not significant. (PDF) [file pone.0054551.s003.pdf]

K85A #8

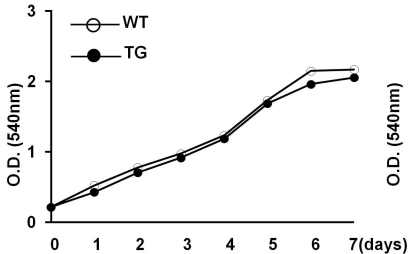

K85A #34

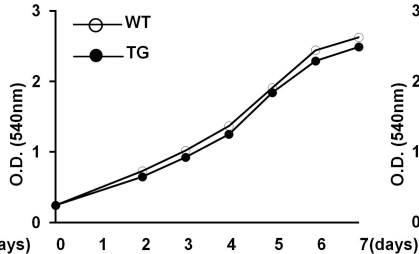

S9A #17

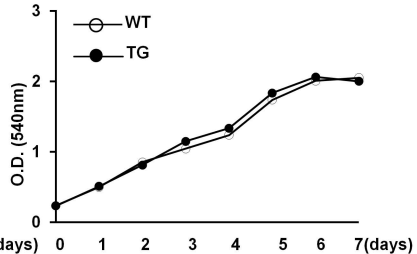

Supplement: Figure S4 — Effect of GSK3β on the proliferation of ADSCs in transgenic mice expressing GSK3β mutants. ADSCs were isolated from either GSK3β-K85A (#8 and #34 line) or GSK3β-S9A (#17 line) Tg mice and WT littermates. ADSCs were maintained in medium supplemented with 10% FCS. Cell proliferation was determined by the MTT assay during 7 days of culture. (PDF) [file pone.0054551.s004.pdf]

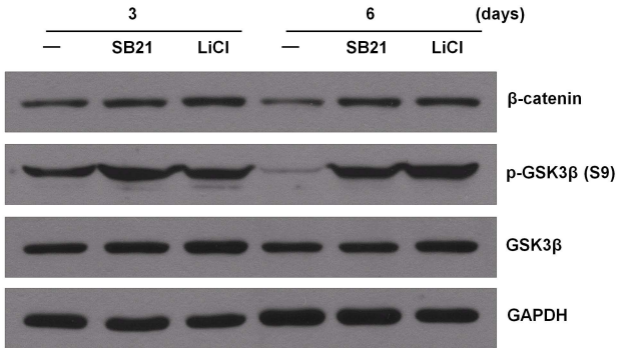

Supplement: Figure S5 — GSK3 inhibitors induce β-catenin accumulation in ADSCs. ADSCs were treated with 5 μΜ SB216763 (SB21) or 20 mM LiCl in osteogenic differentiation medium for the indicated periods of time. Whole cell extracts were immunoblotted with total β-catenin, phospho-Ser9-GSK3β antibody and GSK3β antibody. Membranes were reblotted with GAPDH antibody to ensure equal protein loading. These results are representative of at least three independent experiments. (PDF) [file pone.0054551.s005.pdf]

**A**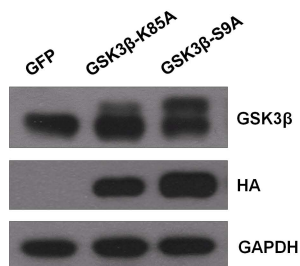**C**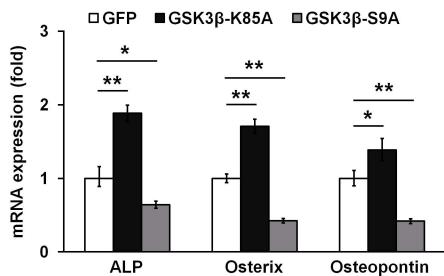**B**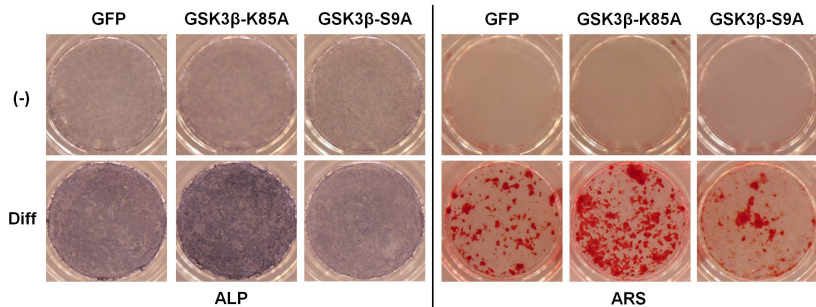**D**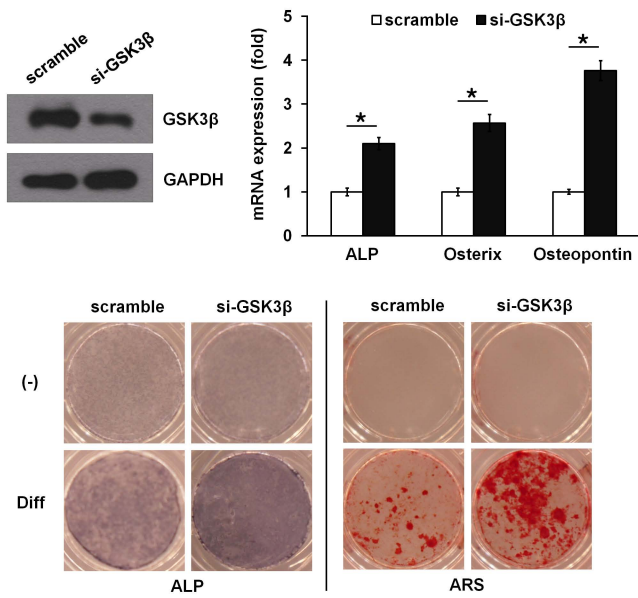

Supplement: Figure S6 — Effects of GSK3β on osteoblast differentiation in BMSCs. (A) BMSCs were infected with either the retrovirus expressing GFP, catalytically inactive GSK3β (GSK3β-K85A) or constitutively active GSK3β (GSK3β-S9A). After 48 hours, cells were harvested for immunoblot analysis for GSK3β expression using antibodies specific for GSK3β or HA. GAPDH served as a loading control. (B) Retroviral infected cells were cultured in osteogenic differentiation medium for 2 weeks. Alkaline phosphatase activity and matrix mineralization were visualized by ALP (ALP) and Alizarin red S (ARS) staining. (C) Total RNA was isolated and analyzed for ALP, osterix, and osteopontin expression by real time PCR. Data represent mean ± S.D. *p<0.05, **p<0.001. Significant differences from the GFP infected group are seen. (D) BMSCs were transiently transfected with 10 nM GSK3β siRNA or a control siRNA (scramble). After 48 hours, GSK3β silencing was examined by immunoblot analysis. GAPDH is used as a loading control. Silencing GSK3β enhanced ALP activity and matrix mineralization as determined by ARS staining. Further, GSK3β silencing increased ALP, osterix and osteopontin mRNA expression as determined by real time PCR analysis. Data represent mean ± S.D. *p<0.001 and show significant difference from scramble siRNA transfected group. (PDF) [file pone.0054551.s006.pdf]

**A**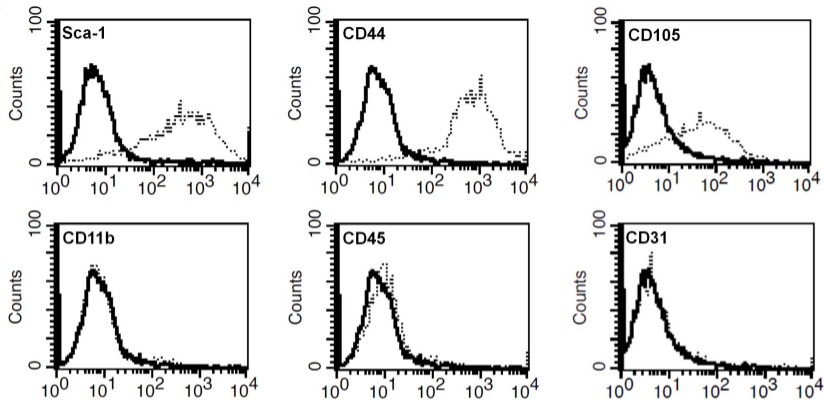**C**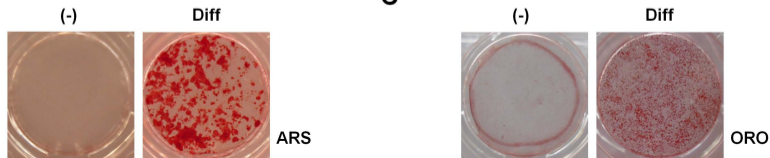

Supplement: Figure S7 — Immunophenotypic characterization and differentiation assays of BMSCs. (A) BMSCs were stained with antibodies against surface markers or control antibodies and subjected to flow cytometry analysis. Cells were homogeneously positive for mesenchymal markers CD44 and CD105 as well as for progenitor cells markers Sca-1 but negative for hematopoietic markers CD11b and CD45 and endothelial markers CD31. The respective isotype control is shown as a thick black-line in the histogram. (B, C) BMSCs were cultured with or without differentiation medium for 2 weeks to induce cell differentiation. Osteoblastogenesis was detected by Alizarin red S (ARS) staining, and adipogenesis was detected by Oil red O (ORO) staining. Original magnification, X 10. (PDF) [file pone.0054551.s007.pdf]

0

2

4

6

8

(days)

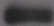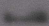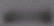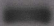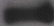

$\beta$ -catenin

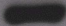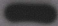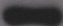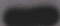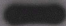

GAPDH

Supplement: Figure S8 — Change of β-catenin levels during osteoblast differentiation. ADSCs were cultured in osteogenic differentiation medium for the indicated periods of time. Whole cell extracts were immunoblotted with total β-catenin. Membranes were reblotted with GAPDH antibody to ensure equal protein loading. These results are representative of at least three independent experiments. (PDF) [file pone.0054551.s008.pdf]
