# Supplementary material for: Glycogen Synthase Kinase 3β Promotes Osteogenic Differentiation of Murine Adipose-Derived Stromal Cells
Source: PLoS One. 2013 Jan 16;8(1):e54551. doi: 10.1371/journal.pone.0054551 (PMC3546989; doi:10.1371/journal.pone.0054551)
Supplement: Methods S1 — More information on the Flow cytometric analysis, BMSC isolation, Adipogenic differentiation and oil red O (ORO) staining, and MicroCT analysis. (DOC) [file pone.0054551.s009.doc]

**Supplementary materials and methods. More information on the Flow cytometric analysis, BMSC isolation, Adipogenic differentiation and oil red O (ORO) staining, and MicroCT analysis.**

**Flow cytometric analysis**

ADSCs were detached from culture dish with trypsin/EDTA and resuspended pellet with 5 % fetal bovine serum (serum-PBS). Centrifuged at 500 x g for 5 minutes and repeat this washing step one more time. About 3 x 10^5^ cells were divided into aliquots in a 1.5 ml microcentrifuge tube, resuspended in 500 μl serum-PBS and pelleted by centrifugation for 5 minutes at 500 x g. The cells were stained with fluorescent isothiocyanate (FITC)-conjugated rat anti–mouse CD44, Sca-1, CD11b and CD45, or PE-conjugated rat anti–mouse CD31 and CD105 (eBioscience, San Diego, CA) at a concentration of 2 μg/ml at 4 °C for 30 minutes. The cells stained with FITC- or PE-conjugated rat anti-mouse IgG served as controls. Then, cells were centrifuged at 800 x g for 5 minutes, washed twice with serum-PBS and fixed with 1 % paraformaldehyde. Cells were resuspended in serum-PBS, examined by FACS Callibur cytometry (Becton Dickinson, San Jose, CA) and analyzed using cell quest software.

**BMSC isolation**

BMSCs were isolated from 3 to 4-week-old C57BL/6 mice (The Jackson Laboratory) as described previously [1]. In brief, mice were sacrificed by cervical dislocation and their tibiae and femurs were carefully cleaned from attached soft tissues. Cells were flushed out from the marrow with α-MEM using a syringe needle inserted into one end of the bone. After washing the bone cavities, tibiae and femurs were cut into chips and digested with 0.1 % collagenase II (Sigma) for 1 hour at 37 °C with vigorous shaking. The digestion medium and released cells were discarded, and the bone chips were placed in cell culture dishes in α-MEM containing 10 % FBS, 100 units/ml penicillin, and 100 μg/ml streptomycin at 37 °C in a 5 % CO_2_ humidified incubator. After 5 days, adherent cells were harvested by trypsinization and further cultured. The cells at passages 2 to 5 were used in all experiments described.

**Adipogenic differentiation and oil red O (ORO) staining**

For adipogenesis, cells were allowed to grow to 70 % to 90 % confluence and then cultured in adipogenic medium containing 50 μg /ml indomethacin (Sigma), 10^-7^ M dexamethasone (Sigma), 5 μg /ml insulin (Sigma) and 10 uM rosiglitazone (Alexis). The culture medium was changed three times per week for 1 to 2 weeks and assessed by the use of an oil red O staining as an indicator of intracellular lipid accumulation.

**MicroCT analysis**

Bones were fixed in 10% formaldehyde, decalcified in 0.5 M ethylenediaminetetraacetic acid (EDTA), pH 7.4. Quantitative microcomputed tomography (μCT) was performed with Skyscan 1076 (Skyscan N.V., Belgium). The data from scanned slices was used for the 3-dimentional analysis to calculate femoral morphometric parameters by CT-AN 1.10 (Skyscan N.V., Belgium). The nomenclature and units were according to the recommendation of the Nomenclature Committee of the American Society for Bone and Mineral Research [2].

**References**

1. Zhu H, Guo ZK, Jiang XX, Li H, Wang XY et al. (2010) A protocol for isolation and culture of mesenchymal stem cells from mouse compact bone. Nat Protoc 5: 550-560.

2. Parfitt AM, Drezner MK, Glorieux FH, Kanis JA, Malluche H, et al. (1987) Bone histomorphometry: standardization of nomenclature, symbols, and units. Report of the ASBMR Histomorphometry Nomenclature Committee. J Bone Miner Res 2:595-610.
